# Assessment of Foodborne Disease Hazards in Beverages Consumed in Nigeria: A Systematic Literature Review

**DOI:** 10.1089/fpd.2021.0043

**Published:** 2022-01-11

**Authors:** David O. Oduori, Emmah Kwoba, Lian Thomas, Delia Grace, Florence Mutua

**Affiliations:** ^1^ International Livestock Research Institute, Nairobi, Kenya.; ^2^ Department of Animal Health and Production, Maasai Mara University, Narok, Kenya.; ^3^ Institute of Infection Veterinary & Ecological Sciences, University of Liverpool, Wirral, United Kingdom.; ^4^ Natural Resource Institute, University of Greenwich, Kent, United Kingdom.

**Keywords:** foodborne disease, hazards, beverages, Nigeria

## Abstract

Risk assessment is a formal process of identifying hazards and assessing the risk associated with them (risk is a combination of the severity of illness and the probability of occurrence). This review highlights foodborne disease hazards reported in beverages consumed in Nigeria for the period between 2000 and 2020. Based on a preregistered protocol and search syntax, studies were retrieved from the PubMed, Google Scholar, and ScienceDirect databases. Rayyan QCRI software was used to screen the articles. Data were then extracted from the included full-text articles, into a standardized excel workbook. A total of 18,762 articles were identified, from which 126 were included in the final analyses. The common beverages studied were sachet water (14.9%), borehole/well water (13.9%), cereal-based beverages (12.1%), raw/fresh milk (8.3%) and *nono/nunu*, which is a fermented milk-cereal beverage (7.2%). Sufficient data were available to undertake pooled prevalence estimates for some hazards within select beverages and revealed contamination rates for *Staphylococcus* spp. in raw/fresh milk, 12.3% (95% CI 6.3–20.0); *Salmonella* spp. in borehole/well water, 19.8% (95% CI 13.1–27.4); *Klebsiella* spp. in sachet water, 40.0% (95% CI 12.4–71.7); *Staphylococcus* spp. in *nono/nunu*, 32.6% (95% CI 14.7–53.8), and *Escherichia* spp. in *nono/nunu*, 30.7% (95% CI 21.9–40.2). Heterogeneity was present in the aggregate summary estimates. This review has highlighted the presence of several hazards of high importance to public health in commonly consumed beverages in Nigeria. The data presented here provide an entry point for future quantitative risk assessments both to determine the level of exposure of the community to these hazards and also for the identification of the most effective mitigation strategies to reduce these risks and improve health outcomes in Nigeria.

## Introduction

Globally, 420,000 deaths and 600 million illnesses were attributed to foodborne diseases caused by infectious agents in 2010 (Havelaar *et al.*, 2015). The highest populous affected was from Africa followed by Southeast Asia, with diarrheal disease agents being implicated as the major contributor, responsible for over 50% of the deaths (Muller and Krawinkel, 2005; Havelaar *et al.*, 2015). Consequently, the global foodborne disease burden is estimated at 33 million Disability Adjusted Life Years (DALYs), 40% of this is borne by children younger than 5 years (Muller and Krawinkel, 2005; Havelaar *et al.*, 2015). Contamination of food by heavy metals is responsible for an estimated 1 million illnesses, over 56,000 mortalities, and a DALY metric of 9 million globally. This estimate considers chemical contamination of food by arsenic, methylmercury, lead, and cadmium only (Gibb *et al.*, 2019).

Food safety has been flagged as a significant barrier toward social and economic development and in the attainment of Sustainable Development Goals 1–3, “No Poverty,” “Zero Hunger,” and “Good Health and Well-being” (Havelaar *et al.*, 2015). The inverse relationship between a decline in economic prosperity and foodborne illness in developing societies suggests that addressing food safety may positively contribute to economic gains (Akhtar *et al.*, 2014). Foodborne disease costs developing countries at least $100 million a year (Jaffee *et al.*, 2020).

Subregion “AFR D,” to which Nigeria belongs, was found to have the highest burden of foodborne illness: 1276 (459–2263) DALYs per 100,000 population (Havelaar *et al.*, 2015). The frequency of illnesses related to the consumption of contaminated food could imply the lack of efficient food safety control systems (Mensah *et al.*, 2012; Paudyal *et al.*, 2017). For Nigeria, this assertion is supported by outputs from anthropometric surveys that report a stunting and malnutrition prevalence of 36.8% and 8.9%, respectively, in children younger than 5 years (NPC and ICF, 2019). Bacterial and parasitic diseases are known risk factors for malnutrition in developing countries (Muller and Krawinkel, 2005). The burden due to aflatoxins has also been shown to be high in some African countries, including Nigeria (Havelaar *et al.*, 2015).

Interventions to address hazards in foods are urgently needed. Determining what strategies can be used, alongside other key health burdens across society, should, however, be done in a rational and, where possible, objective manner. It is not only the burden of disease attributable to pathogens or products that should be considered, but also the cost-effectiveness or net economic benefit of potential control interventions. The initial process in such systematic prioritization processes is a risk analysis where, in a stepwise manner, hazards and the population at risk are identified (risk ranking), a quantification (or semiquantitative evaluation) of the risk posed by the hazards is made (risk assessment), risk mitigation steps are identified (risk management), and finally, risk communication is undertaken. This study contributes to the first steps of risk ranking and risk assessment by identifying foodborne hazards of potential risk to Nigerian consumers. Many foodborne pathogens can be found in common beverages, with water being a well-known example of a beverage with a high burden of disease attributable to the pathogens it may carry. Beverages consumed in Nigeria have been reported to harbor various hazards and are thus a public health concern.

A Systematic Literature Review (SLR) was undertaken to establish the current evidence on foodborne hazard occurrence in a myriad of beverages consumed in Nigeria between the year 2000 and the year 2020. The information will inform further risk assessment work in Nigeria. It will assist in determining the riskiest beverage products, potential mitigation activities, and their impacts. This review, therefore, highlights hazards associated with commonly consumed beverages in Nigeria. The findings will inform the scope of further risk assessment work in foods in formal and informal markets in Nigeria, and other countries with similar situations.

## Materials and Methods

### Protocol development

A protocol to guide the SLR process was developed and registered in PROSPERO and is searchable in https://www.crd.york.ac.uk/prospero/ using CRD42020184768 as the registration number.

### Literature search

Searches were done in three search engines, namely PubMed, Science Direct, and Google Scholar. The search was set to identify articles published in the period 2000–2020. An initial syntax covering most beverages and safety terms was developed for PubMed using Medical Subject Headings (MeSH) terms and Boolean search operators (Supplementary Data S1). Since ScienceDirect has a syntax string limit of 8 Boolean expressions, therefore a series of 40 short syntaxes, including all the beverage and safety terms in the PubMed syntax, was developed. These articles were exported to the Mendeley reference manager from where duplicates were identified and removed. The resulting unique file was treated as a single output from ScienceDirect. Similarly, for Google Scholar, which has a character limit of 256, a series of 20 syntaxes was developed to include all the beverage and safety terms in the syntax used in PubMed. For each search output, the first 300 hits were considered for the review (Haddaway *et al.*, 2015) and were exported to Mendeley via its Web Importer function. All the search outputs were pooled in the Mendeley reference manager, and the duplicates were identified and removed, resulting in a unique Google Scholar file.

### Article screening

This review included observational studies published between 2000 and 2020. Only studies conducted in Nigeria and those published in English were included in the review. Studies with experimental design, on water quality/safety not associated with drinking water, antimicrobial resistance, and those not considering biological or chemical hazards associated with beverage consumption were excluded.

The screening process was facilitated by the Rayyan QCRI (https://rayyan.qcri.org/), a web-based and mobile-based application that is used for screening articles in SLRs (Ouzzani *et al.*, 2016). The search outputs from the three databases were exported (directly in the case of PubMed and from Mendeley in the case of ScienceDirect and Google Scholar) to the software where any duplicates were identified and subsequently resolved. Publication titles and abstracts were then screened against the inclusion and exclusion criteria as specified in the preregistered protocol. Four reviewers participated in the process. The screening was done independently by two reviewers, Reviewers 1 and 2. Articles were then subjected to full-text screening against the predetermined criteria and reasons for the exclusion provided. Articles that were found acceptable after the full-text screening were considered for data extraction. Any discordance in the classification of articles by Reviewers 1 and 2 was resolved by Reviewers 3 and 4, and this applied for both abstract and full article screening. For quality control, 5% of the included and 5% of the excluded articles were reviewed by Reviewers 3 and 4. Additional articles were identified through screening of the reference section of the included publications. The output of the screening process was then reported according to the PRISMA 2009 flow diagram guidelines (Moher *et al.*, 2015).

### Quality assessment and data extraction

Data extraction and quality assessment happened concurrently. Quality assessment of individual articles was based on the Cochrane assessment of bias (Higgins *et al.*, 2021). The articles were classified as being of good, medium, and poor quality. Articles that had an unbiased selection of subjects, where the methods were judged to be scientifically sound, and appropriate data analysis had been conducted with complete and accurate results were judged as good quality. Medium-quality articles had acknowledged and accounted for selection bias of subjects, limitations in data analysis, understandable methods, and valid results. Articles that did not acknowledge selection bias of subjects had incomplete and inaccurate methods, inappropriate data analysis, and incomplete results judged to be of poor quality were excluded from the study, and data were not extracted from them.

A data extraction template in Microsoft Excel was developed and piloted. The template was designed to capture the following data: article identifiers (article ID, the title of the publication, authors, year of publication), type of study, geographical locality, name of the beverage, name and category of hazard, point of sampling, sampling technique, specific laboratory test used, number of samples analyzed, number positive for the hazard, and raw data on the concentration of hazards. For review articles, data were not extracted directly from them but from the primary data, articles cited.

### Data management and analysis

The data were cleaned and validated to check for any errors and omissions. They were analyzed using the R platform for statistical computing software (R Core Team, 2018). Data on type of beverage, type of study, category, and specific hazards, point and method of sampling, and laboratory test used were summarized as proportions and presented as tables and figures. A random-effects model was selected for estimating pooled prevalence due to the interstudy differences (Bown and Sutton, 2010). To ascertain stronger statistical inference, pooled prevalence was estimated for hazard/beverage combinations where 5≥ good-quality studies were available (Jackson and Turner, 2017). Forest plots and summary tables including estimates of heterogeneity were generated using the metaviz package of R software. Heterogeneity thresholds were derived from Deeks *et al.* (2019), where *I*^2^ of 0–40% represented low heterogeneity or homogeneity.

## Results

### Database search output and screening

**FIG. 1.**
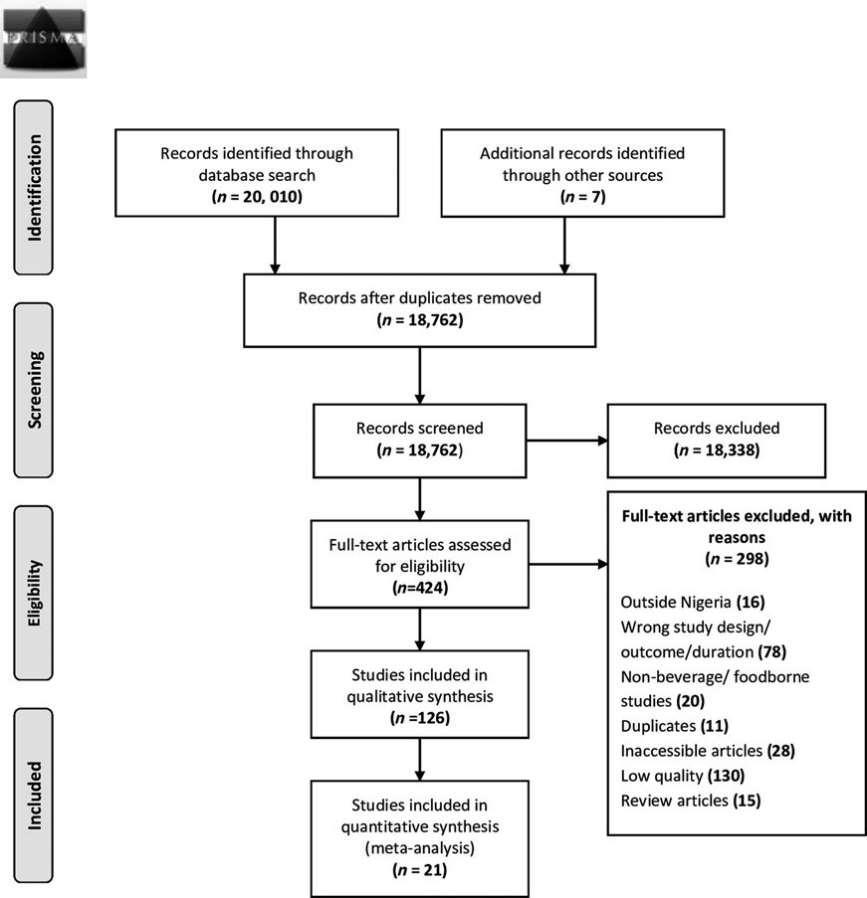
PRISMA 2009 flow diagram occurrence of FBD hazards in beverages consumed in Nigeria. FBD, foodborne disease. From Moher *et al.* (2009). Fore more information, visit www.prisma-statement.org

A total of 20,100 articles were retrieved from the 3 databases: PubMed (1315), ScienceDirect (14,406), and Google Scholar (4289). Seven additional articles were identified from review articles. A total of 1255 duplicates were removed (either in Mendeley or in Rayyan QCRI). After title and abstract screening, 18,338 articles were excluded, resulting in 424 articles that were subjected to full-text screening. Two hundred ninety-eight articles were excluded at the stage of full article review on the following basis: low quality (130), wrong study design or outcome (77), inaccessible articles where the full text was not available (28), nonbeverage or nonfoodborne studies (20), articles with a geographical focus outside Nigeria (16), review articles (15), additional duplicates not previously identified (11), and articles outside the time frame of interest (1). A total of 126 articles were thus included in the qualitative analyses, of which 21 articles were included in pooled prevalence analysis. The PRISMA 2009 flowchart is given in Figure 1.

### Characteristics of articles included in the qualitative synthesis

The 126 articles included in the qualitative analyses resulted in a total of 787 records. Among the 126 articles, 73.8% had some details lacking in the methods section, but bias in the selection of subjects and limitations in data analysis had been acknowledged and the articles were therefore deemed to be of medium quality. Only 26.2% of the articles were judged to be of good quality, as they had an unbiased selection of subjects, scientifically sound study methods, appropriate data analysis, and complete and accurate results. The majority (76.2%) of the studies retrieved had been published between 2010 and 2018. Sampling was done at the point of retail/trader (57.6%), processing plant (3.8%), or source/farm/point of harvest/manufacture (29.6%). Nine percent of the studies did not specify the point at which sampling was conducted.

The distribution of the studies was varied. Notably, the following states Kebbi, Kwara, Adamawa, Yobe, Zamfara, Jigawa, Kogi, and Katsina were not represented in the reviewed literature. The selected studies covered 28 of the 36 federal states of Nigeria.

### Beverage types reported in the review

The most common beverages studied (*n* = 787) were sachet water (14.9%), borehole/well water (13.9%), cereal-based beverages (12.1%), raw/fresh milk (8.3%), and *nono/nunu* (7.2%). Other beverage types included river/canal/stream water, processed nonalcoholic beverages, fruit juice, traditional alcoholic beverages, soya milk/tigernut, bottled water, tap/piped water, traditional nonalcoholic drinks, processed milk, processed alcoholic beverages, and rainwater (Table 1).

**Table 1.** Number of Studies Investigating the Contamination of Specific Beverages (Total 787)

Name of beverage	Number of studies identified (%)	Name of beverage	Number of studies identified (%)
Sachet water	117 (14.9)	Yoghurt	33 (4.2)
Borehole/well water	109 (13.9)	Fermented milk^a^	25 (3.2)
Cereal-based beverage^b^	95 (12.1)	Soya milk/tigernut	18 (2.3)
Raw/fresh milk	65 (8.3)	Bottled water	18 (2.3)
*Nono/nunu*	57 (7.2)	Tap/piped water	16 (2)
Zobo	46 (5.8)	Traditional nonalcoholic drinks^c^	14 (1.8)
River/Canal/Stream	43 (5.5)	Processed milk	9 (1.1)
Processed nonalcoholic^d^	35 (4.4)	Processed alcoholic^e^	9 (1.1)
Fruit juice	35 (4.4)	Rainwater	8 (1)
Traditional alcoholic^f^	35 (4.4)		

^a^ For example, Kesham, Kindrimo, Manshanu, Wara.

^b^ Kunu, Kunun-zaki, Kunu-aya, Akamu, Ogi.

^c^ For example, herbal teas.

^d^ Canned and noncanned beverages, soft drinks.

^e^ For example, beer.

^f^ For example, Burukutu, Gin Ufofop, Pito.

### Hazards reported in the studied beverages

The 5 main hazard groups reported in this review include bacteria, fungi, parasites, biotoxins, and chemical contaminants (a total of 754 records). The most common hazards, or hazard proxies, studied were bacterial (483 records, 64.1%) and chemical contaminants (179 records, 23.7%). A few studies on fungi (55 records, 7.3%), toxins (24 records, 3.2%), and parasites (13 records, 1.7%) were identified. Thirty-three records did not specify the identity of the hazard. The most often studied bacterial genus (*n* = 483) was *Escherichia* spp. (16.1%, six studies had negative findings), *Staphylococcus* spp. (14.3%, one study had a negative finding), *Salmonella* spp. (11%, two studies had negative findings), coliforms (8.7%, two studies had negative findings), and *Klebsiella* spp. (6.6%, one study had negative findings). The common fungal and parasitic contaminants studied and reported were as follows (*n* = 55; *n* = 13, respectively): *Aspergillus* spp. (27.3%), *Penicillium* spp. (14.5%), *Rhizopus* spp. (12.7%), *Giardia* spp. (38.5%), *Entamoeba* spp. (30.8%), and *Ascaris* spp. (23.1%).

Commonly studied chemical contaminants (*n* = 179) were lead (14.5%, seven reports had negative findings), iron (8.9%, seven studies had negative findings), and chromium (8.4%, six studies had negative findings). The fungal metabolites (*n* = 24) investigated included acetyl-deoxynivalenol/deoxynivalenol (33.3%), zearalenone (33.3%, three reports had negative findings), and fumonisin (25%). See Tables 2–5 for more details.

**Table 2.** Hazards Reported in Different Types of Beverages in Nigeria Between the Years 2000–2020

Name/type of beverage	Hazard group	Name of pathogens (genus)/chemicals	Reference
Cereal-based beverages (Kunu, Kunun-zaki, Kunu-aya, Akamu, Ogi) Kunu/Kunun-zaki/Kunu-aya is a Cereal-based nonalcoholic fermented beverage, produced by steeping of sorghum, millet, or maize, wet milling, sieving, and partial gelatinization of the slurry (Ndulaka *et al.*, 2014). Akamu is a porridge made from fermented maize, millet, or sorghum. It is used for weaning infants and taken as breakfast (Nwokoro and Chukwu, 2012) and Ogi is a fermented cereal pudding (Olasupo *et al.*, 2002)	Bacterial	*Bacillus* spp., *Citrobacter* spp., *Coliforms*, *Enterobacter* spp., *Enterococcus* spp., *Escherichia* spp., *Klebsiella* spp., *Lactobacillus* spp., *Listeria* spp., *Proteus* spp., *Pseudomonas* spp., *Shigella* spp., *Salmonella* spp., *Staphylococcus* spp., *Streptococcus* spp.	Olasupo *et al.* (2002), Umoh *et al.* (2004), Nwachukwu *et al.* (2009), Ikpoh *et al.* (2013), Makut *et al.* (2013), Aboh and Oladosu (2014), Umaru *et al.* (2014), Zumbes *et al.* (2014), Etang *et al.* (2017), Katuka *et al.* (2018), Ogodo *et al.* (2018), Popoola *et al.* (2019)
	Fungal	*Aspergillus* spp., *Candida* spp., *Fusarium* spp., *Penicillium* spp., *Saccharomyces* spp., *Rhizopus* spp.	Ejiogu *et al.* (2010), Ikpoh *et al.* (2013), Aboh and Oladosu (2014), Etang *et al.* (2017)
	Chemical	Chromium, iron, lead, zinc	Bakare-Odunola and Mustapha (2014), Maigari *et al.* (2016)
Fermented milk (Kesham, Kindrimo, Manshanu, Wara)	Bacterial	Coliforms, *Escherichia* spp., *Klebsiella* spp., *Salmonella* spp., *Shigella* spp., *Staphylococcus* spp., *Streptococcus* spp.	Olasupo *et al.* (2002), Ogbonna *et al.* (2012), Karshima *et al.* (2013), Makut *et al.* (2014b), Umaru *et al.* (2014), Fowoyo (2016), Musa *et al.* (2017), Aliyu *et al.* (2018, 2020)
*Nono/Nunu* *Nono/Nunu* is crude cultured whole milk (Onyinye *et al.*, 2020).	Bacterial	*Bacillus* spp., coliforms, *Escherichia* spp., *Klebsiella* spp., *Mycobacterium* spp., *Pseudomonas* spp., *Salmonella* spp., *Shigella* spp., *Staphylococcus* spp., *Streptococcus* spp.	Olasupo *et al.* (2002), Ofukwu *et al.* (2008), Okonkwo (2011), Yabaya *et al.* (2012), Karshima *et al.* (2013), Reuben and Owuna (2013), Agada *et al.* (2014), Egwaikhide *et al.* (2014), Ivbade *et al.* (2014), Makut *et al.* (2014a), Enem *et al.* (2015), Enabulele and Nwankiti (2016), Fowoyo (2016), Okpo *et al.* (2016), Usman and Mustapha (2016), Musa *et al.* (2017), Dafur *et al.* (2018), Onioshun (2018), Yakubu *et al.* (2018), Aliyu *et al.* (2020)
	Fungal	*Trichoderma* spp., *Aspergillus* spp., *Mucor* spp., *Candida* spp.	Okonkwo (2011), Egwaikhide *et al.* (2014), Dafur *et al.* (2018)
	Chemical	Antimicrobial residues	Okonkwo (2011), Onyinye *et al.* (2020)
Processed milk (pasteurized milk)	Bacterial	*Klebsiella* spp., *Salmonella* spp., *Shigella* spp., *Staphylococcus* spp., *Staphylococcus* spp., *Streptococcus* spp.	Umaru *et al.* (2014), Dayok *et al.* (2019)
	Chemical	Antimony, mercury, tin	Roberts and Orisakwe (2011)
Raw/fresh milk	Bacterial	Coliforms (not further described), *Escherichia* spp., *Klebsiella* spp., *Lactobacillus* spp., *Listeria* spp., *Micrococcus* spp., *Mycobacterium* spp., *Proteus* spp., *Pseudomonas* spp., *Salmonella* spp., *Shigella* spp., *Staphylococcus* spp., *Campylobacter* spp., *Cellulomonas* spp., *Citrobacter* spp., *Streptococcus* spp., *Xanthomonas* spp., *Bacillus* spp.	Ofukwu *et al.* (2008), Cadmus *et al.* (2010), Salihu *et al.* (2010), Adesina *et al.* (2011), Ogbonna *et al.* (2012), Enurah *et al.* (2013), Karshima *et al.* (2013), Agada *et al.* (2014), Ivbade *et al.* (2014), Makut *et al.* (2014a), Oluduro *et al.* (2014), Umaru *et al.* (2014), Enem *et al.* (2015), Mailafia *et al.* (2017), Onioshun (2018), Yakubu *et al.* (2018), Dayok *et al.* (2019), Aliyu *et al.* (2020)
	Chemical	Aflatoxin M1&M2, antimicrobial residues	Chilaka *et al.* (2018), Onyinye *et al.* (2020)
Yoghurt	Bacterial	*Aeromonas* spp., *Bacillus* spp., *Clostridium* spp., Coliforms, *Escherichia* spp., *Klebsiella* spp., *Lactobacillus* spp., *Pseudomonas* spp., *Salmonella* spp., *Staphylococcus* spp., *Streptococcus* spp.	Nwagu and Amadi (2010), Mbaeyi-Nwaoha and Egbuche (2012), Makut *et al.* (2014b), Oluduro *et al.* (2014), Umaru *et al.* (2014), Chukwu *et al.* (2015), Enem *et al.* (2015), Sunday *et al.* (2016), Usman and Mustapha (2016)
	Fungal	*Fusarium* spp., *Geotrichum* spp., *Neurospora* spp., *Penicillium* spp., *Aspergillus* spp., *Absidia* spp.	Sunday *et al.* (2016)
Soya milk/tigernut	Bacterial	*Bacillus* spp., Coliforms, *Escherichia* spp., *Lactobacillus* spp., *Salmonella* spp., *Staphylococcus* spp., *Streptococcus* spp.	Brooks *et al.* (2003), Anagu *et al.* (2015), Ntukidem *et al.* (2020)
	Fungal	*Aspergillus* spp., *Rhizopus* spp.	Brooks *et al.* (2003), Ntukidem *et al.* (2020)
Processed alcoholic beverages (Beer)	Chemical	Aluminum, cadmium, copper, iron, lead, zinc	Udota and Umoudofia (2011), Jegede *et al.* (2016), Okareh *et al.* (2018)
Processed nonalcoholic (canned and noncanned beverages, soft drinks)	Chemical	Antimony, arsenic, cadmium, chromium, copper, fluoride, iron, lead, manganese, mercury, nickel, tin, zinc	Maduabuchi *et al.* (2007, 2008), Roberts and Orisakwe (2011), Adepoju-Bello *et al.* (2012), Godwill *et al.* (2015), Magomya *et al.* (2015), Jegede *et al.* (2016), Ani *et al.* (2020)
	Fungal	*Mucor* spp., *Mycelia* spp., *Aspergillus* spp., *Penicillium* spp., *Rhizopus* spp., *Saccharomyces* spp., *Schizosaccharomyces* spp.	Oyetunji (2006)
Traditional alcoholic drinks (Burukutu, Gin Ufofop, Pito)	Chemical	Antimony, arsenic, cadmium, nickel, chromium, copper, fluoride, iron, lead, manganese, mercury, tin, zinc	Udota and Umoudofia (2011), Chilaka *et al.* (2018)
	Fungal	*Saccharomyces* spp., *Aspergillus* spp., *Mucor* spp., *Schizosaccharomyces* spp., *Mycelia* spp., *Rhizopus* spp., *Penicillium* spp.	Olaniyi and Akinyele (2020)
	Toxins	Acetyl-deoxynivalenol, fumonisins B1, B2, B3, B4 and zearalenone	Chilaka *et al.* (2018)
Traditional nonalcoholic drinks (herbal tea)	Bacterial	*Escherichia* spp., *Bacillus* spp., *Klebsiella* spp., *Pseudomonas* spp., *Salmonella* spp., *Staphylococcus* spp.	Omogbai and Ikenebomeh (2013)
	Fungal	*Aspergillus* spp., *Fusarium* spp., *Penicillium* spp., *Rhizopus* spp., *Serratia* spp.	Omogbai and Ikenebomeh (2013)
*Zobo* Zobo is a hot water extract of Hibiscus sabdariffa (Ndulaka *et al.*, 2014)	Parasitic	*Trichuris* spp., *Giardia* spp., *Ascaris* spp., *Entamoeba* spp., *Balantidium* spp.	Ekwunife *et al.* (2014)
	Fungal	*Fusarium* spp., *Aspergillus* spp., *Penicillium* spp., *Rhizopus* spp., *Saccharomyces* spp.	Oku *et al.* (2018), Onuorah and Odibo (2018)
	Chemical	Zinc, lead, chromium, iron	Bakare-Odunola and Mustapha (2014), Anagu *et al.* (2015), Maigari *et al.* (2016)
	Bacterial	*Bacillus* spp., *Clostridium* spp., Coliforms, *Shigella* spp., *Corynebacterium* spp., *Escherichia* spp., *Klebsiella* spp, *Lactobacillus* spp., *Proteus* spp., *Salmonella* spp., *Staphylococcus* spp., *Streptococcus* spp.	Mbaeyi-Nwaoha and Egbuche (2012), Risiquat (2013), Ejikeugwu *et al.* (2014), Umaru *et al.* (2014), Zumbes *et al.* (2014), Anagu *et al.* (2015), Ezeigbo *et al.* (2015)
Fruit Juice	Bacterial	*Acetobacter* spp., *Alicyclobacillus* spp., *Bacillus* spp., *Enterobacter* spp., *Escherichia* spp., *Klebsiella* spp., *Lactobacillus* spp., *Staphylococcus* spp., *Streptococcus* spp.	Agwa *et al.* (2014), Maduka *et al.* (2014), Ogodo *et al.* (2016), Osopale *et al.* (2016)
	Fungal	*Penicillium* spp., *Rhizopus* spp., *Saccharomyces* spp., *Aspergillus* spp.	Agwa *et al.* (2014), Ogodo *et al.* (2016)
	Chemical	Zinc, copper, lead	Okeri *et al.* (2009)
Rainwater	Bacterial	*Enterobacter* spp., *Escherichia* spp., *Klebsiella* spp., *Proteus* spp., *Salmonella* spp., *Shigella* spp., *Streptococcus* spp., *Vibrio* spp.	Charity *et al.* (2012)
River/Canal/Stream water	Bacterial	Coliforms, *Enterobacter* spp., *Escherichia* spp., *Klebsiella* spp., *Proteus* spp., *Pseudomonas* spp., *Salmonella* spp., *Shigella* spp., *Staphylococcus* spp., *Streptococcus* spp., *Vibrio* spp.	Iroegbu *et al.* (2000), Shittu *et al.* (2008), Charity *et al.* (2012)
	Chemical	Lead, nickel, nitrate, nitrite, phosphate, alkalinity, cadmium, chromium, copper, zinc	Duruibe *et al.* (2007), Adefemi and Awokunmi (2010), Nduka *et al.* (2010), Yahaya *et al.* (2012)
Sachet water	Chemical	Lead, magnesium, manganese, molybdenum, PPCPs, nickel, selenium, arsenic, bicarbonate, cadmium, bromine, calcium, chloride, chromium, copper, iron, fluoride, iron, zinc	Oboh *et al.* (2001), Orisakwe *et al.* (2006), Okeri *et al.* (2009), Raji *et al.* (2010), Abua *et al.* (2012), Ani *et al.* (2020), Ebele *et al.* (2020)
	Bacterial	*Micrococcus* spp., *Proteus* spp., *Vibrio* spp., *Salmonella* spp., *Pseudomonas* spp., *Serratia* spp., *Shigella* spp., *Staphylococcus* spp., *Streptococcus* spp., *Bacillus* spp., *Klebsiella* spp., *Chromobacterium* spp., coliforms *Enterobacter* spp., *Escherichia* spp., *Aeromonas* spp., *Flavobacterium* spp., *Acinetobacter* spp., *Alcaligenes* spp.	Oboh *et al.* (2001), Ajayi *et al.* (2008), Ezeugwunne *et al.* (2009), Olaoye and Onilude (2009), Mudasiru *et al.* (2011), Ngwai *et al.* (2010), Akinyemi *et al.* (2011), Mgbakor *et al.* (2011), Mbaeyi-Nwaoha and Egbuche (2012), Oluwafemi and Oluwole (2012), Onilude *et al.* (2013), Isikwue and Chikezie (2014), Ugochukwu *et al.* (2015), Okunola *et al.* (2018)
	Parasitic	*Ascaris* spp., *Trichuris* spp., *Entamoeba* spp., *Giardia* spp., *Ancylostoma/Necator* spp.	Ekwunife *et al.* (2010), Omolade *et al.* (2017)
Borehole/well water	Bacterial	*Proteus* spp., *Pseudomonas* spp., *Salmonella*, *Shigella* spp., *Staphylococcus* spp., *Vibrio spp. Streptococcus* spp., *Coliforms*, *Klebsiella* spp., *Flavobacterium* spp., *Campylobacter* spp, *Enterobacter* spp., *Escherichia* spp.	Ibe and Okplenye (2005), Duruibe *et al.* (2007), Shittu *et al.* (2008), Mudasiru *et al.* (2011), Raji *et al.* (2010), Agbalagba *et al.* (2011), Akinyemi *et al.* (2011), Eruola *et al.* (2011), Charity *et al.* (2012), Bello *et al.* (2013), Onwughara *et al.* (2013), Ugboma *et al.* (2013), Aboh *et al.* (2015), Lovet and Dineebimo (2017)
	Chemical	Mercury, molybdenum, nickel, nitrate and nitrite, PPCPs, phosphate, potassium, selenium, sodium, zinc, calcium, chromium, copper, fluoride, iron, lead, manganese, alkalinity, arsenic, bromine, cadmium	Duruibe *et al.* (2007), Adefemi and Awokunmi (2010), Mudasiru *et al.* (2011), Nduka *et al.* (2010), Agbalagba *et al.* (2011), Eruola *et al.* (2011), Adindu *et al.* (2012), Bello *et al.* (2013), Egbinola and Amanambu (2014), Ebele *et al.* (2020)
Bottled water	Bacterial	*Escherichia* spp., *Klebsiella* spp., *Pseudomonas* spp., *Staphylococcus* spp., *Streptococcus* spp., coliforms	Ajayi *et al.* (2008), Igbeneghu and Lamikanra (2014)
	Chemical	Antimony, tin, zinc, lead, iron, chromium copper, fluoride, manganese, mercury, tin, zinc, PPCPs	Okeri *et al.* (2009), Roberts and Orisakwe (2011), Ani *et al.* (2020), Ebele *et al.* (2020)
Tap/piped water	Chemical	Arsenic, bromine, chromium, lead, manganese, selenium	Orewole *et al.* (2007), Raji *et al.* (2010)
	Bacterial	Coliforms, *Escherichia* spp., *Salmonella* spp., *Shigella* spp.	Iroegbu *et al.* (2000), Mudasiru *et al.* (2011), Akinyemi *et al.* (2011), Isikwue and Chikezie (2014)

PPCPs, pharmaceuticals and personal care products.

**Table 3.** Number of Studies Investigating Bacterial Contamination of Specific Beverages (Total 483)

Bacteria (genus)	Number of studies (%)	Bacteria (genus)	Number of studies (%)	Bacteria (genus)	Number of studies (%)	Bacteria (genus)	Number of studies (%)
*Escherichia* spp.	78 (16.1)	*Shigella* spp.	24 (5)	*Micrococcus* spp.	5 (1)	*Enterococcus* spp.	2 (0.4)
*Staphylococcus* spp.	69 (14.3)	*Proteus* spp.	21 (4.3)	*Campylobacter* spp.	4 (0.8)	*Aeromonas* spp.	2 (0.4)
*Salmonella* spp.	53 (11)	*Enterobacter* spp.	14 (2.9)	*Citrobacter* spp.	4 (0.8)	*Alcaligenes* spp.	1 (0.2)
*Coliforms*	42 (8.7)	*Alicyclobacillus* spp.	12 (2.5)	*Aeromonas* spp.	4 (0.8)	*Acetobacter* spp.	1 (0.2)
*Klebsiella* spp.	32 (6.6)	*Lactobacillus* spp.	9 (1.9)	*Chromobacterium* spp.	3 (0.6)	*Acinetobacter* spp.	1 (0.2)
*Streptococcus* spp.	26 (5.4)	*Listeria* spp.	7 (1.4)	*Clostridium* spp.	3 (0.6)	*Xanthomonas* spp.	1 (0.2)
*Pseudomonas* spp.	25 (5.2)	*Vibrio* spp.	7 (1.4)	*Serratia* spp.	3 (0.6)	*Corynebacterium* spp.	1 (0.2)
*Bacillus* spp.	25 (5.2)	*Mycobacterium* spp.	6 (1.2)	*Flavobacterium* spp.	2 (0.4)		

**Table 4.** Number of Studies Identified Investigating Parasitic (Total 13) and Fungal (Total 55) Contamination of Specific Beverages

Parasite (genus)	Number of studies (%)	Fungus (genus)	Number of studies (%)	Fungus (genus)	Number of studies (%)
*Giardia* spp.	5 (38.5)	*Aspergillus* spp.	15 (27.3)	*Mucor* spp	2 (3.6)
*Entamoeba* spp.	4 (30.8)	*Penicillium* spp.	8 (14.5)	*Absidia* spp.	1 (1.8)
*Ascaris* spp.	3 (23.1)	*Rhizopus* spp.	7 (12.7)	*Geotrichum* spp.	1 (1.8)
*Trichuris* spp.	2 (15.4)	*Fusarium* spp.	6 (10.9)	*Mycelia* spp.	1 (1.8)
*Balantidium* spp.	1 (7.7)	*Saccharomyces* spp.	6 (10.9)	*Neurospora* spp.	1 (1.8)
*Necator* spp.	1 (7.7)	*Candida* spp.	5 (9.1)	*Schizosaccharomyces* spp.	1 (1.8)
				*Trichoderma* spp.	1 (1.8)

**Table 5.** Number of Studies Identified Investigating Chemical (Total 179) and Mycotoxin (Total 24) Contamination of Beverages

Chemical	Number of studies (%)	Chemical	Number of studies (%)	Chemical	Number of studies (%)	Toxin	Number of studies (%)
Lead	26 (14.5)	Fluoride	5 (2.8)	Tin	3 (1.7)	Acetyl-deoxynivalenol/deoxynivalenol	8 (33.3)
Iron	16 (8.9)	Mercury	5 (2.8)	Alkalinity	3 (1.7)	Zearalenone	8 (33.3)
Chromium	15 (8.4)	Antimony	3 (1.7)	Aluminum	2 (1.1)	Fumonisin	6 (25)
Copper	13 (7.3)	Carbonate	3 (1.7)	Antimicrobial residues	2 (1.1)	Aflatoxin	2 (8.3)
Zinc	12 (6.7)	Bromine	3 (1.7)	Chloride	2 (1.1)		
Cadmium	11 (6.1)	Calcium	3 (1.7)	Magnesium	2 (1.1)		
Manganese	10 (5.6)	Molybdenum	3 (1.7)	Potassium	1 (0.6)		
Nickel	10 (5.6)	PPCPs	3 (1.7)	Sodium	1 (0.6)		
Arsenic	9 (5)	Phosphate	3 (1.7)				
Nitrate/Nitrite	7 (3.9)	Selenium	3 (1.7)				

PPCPs, pharmaceuticals and personal care products.

### Pooled prevalence estimates

**Fig. 2.**
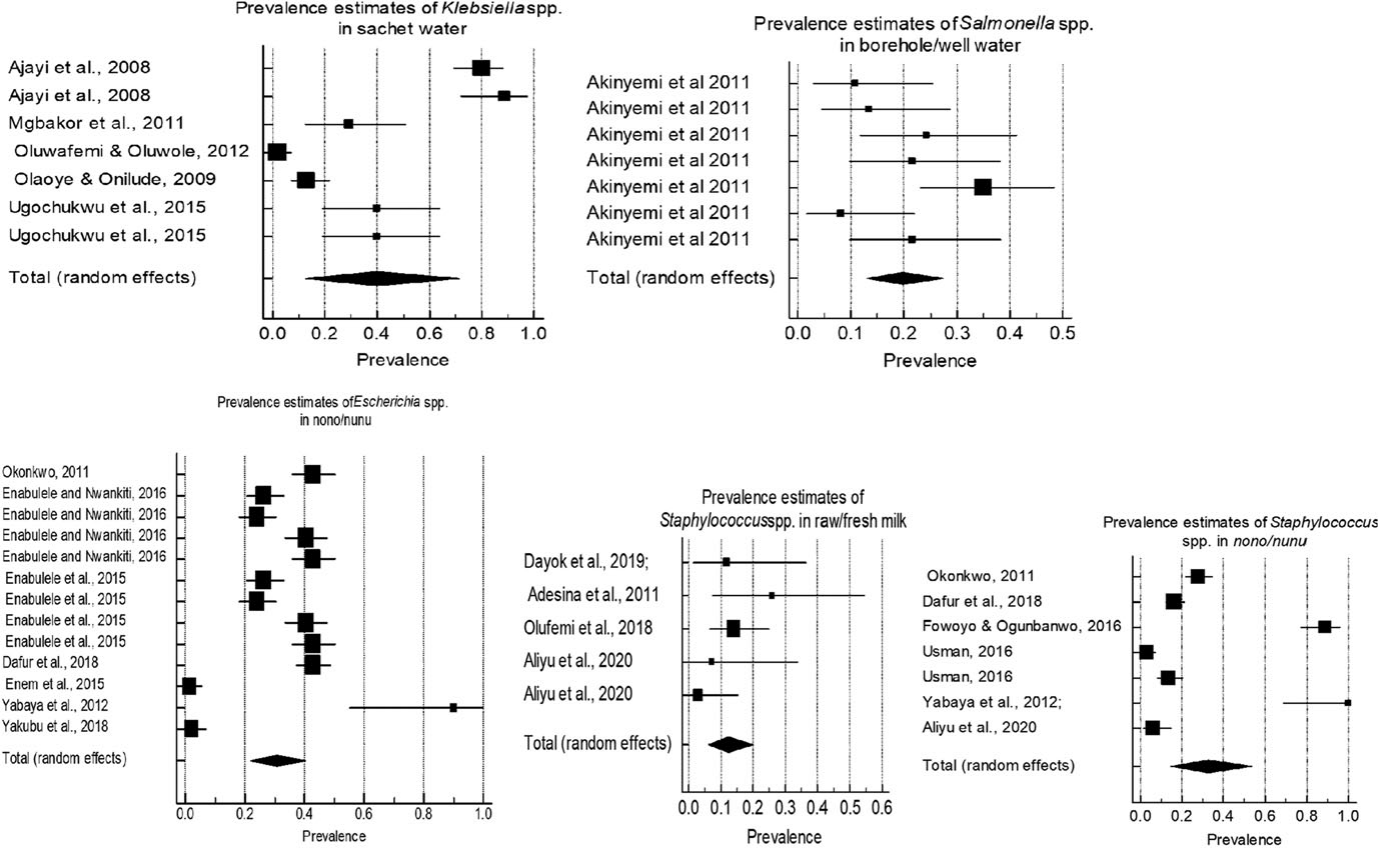
Forest plots of 5 hazard/beverage combinations showing hazard prevalence estimates in individual studies and the pooled prevalence (random effects model). *Staphylococcus* spp. in raw/fresh milk pool was homogenous. *Salmonella* spp. in borehole/well water, pool had moderate heterogeneity. Heterogeneity was considerable in all the other hazard/beverage combinations as denoted by the non-overlapping confidence intervals of the prevalence estimate in several individual studies in these pools.

Sufficient data were available to allow estimation of pooled prevalence for four hazards in four different beverage types (as described in Table 6). Key hazard/beverage combinations with sufficient data (>5 high-quality articles) available to undertake this included *Klebsiella* spp. in sachet water, 40.0% (95% CI 12.4–71.7), *I*^2^ = 97.3%; *Staphylococcus* spp. in *nono/nunu*, 32.6% (95% CI 14.7–53.8), *I*^2^ = 97.5%; and *Escherichia* spp. in *nono/nunu*, 30.7% (95% CI 21.9–40.2), *I*^2^ = 95.7%. *Salmonella* spp. in borehole/well water, 19.8% (95% CI 13.1–27.4), *I*^2^ = 58.4%-moderate heterogeneity and *Staphylococcus* spp. in raw/fresh milk, 12.3% (95% CI 6.3–20.0), *I*^2^ = 34.22%. Heterogeneity based on the *I*^2^ statistic was moderate-considerable in 4/5 hazard/beverage pools. The aggregate summary estimates from these studies are therefore to be interpreted with caution. Only one pool was homogenous, and that was *Staphylococcus* spp. in raw/fresh milk. Forest plots illustrating the data are shown in Figure 2.

**Table 6.** Pooled Prevalence Estimates of Select Biological Hazards in Beverages

Hazard	Beverage	Study	Sample size	Prevalence (%)	95% CI	Weight (%) random effects model
*Klebsiella* spp.	Sachet water	Ajayi *et al.* (2008)	78	80.0	69.4–88.2	14.58
		Ajayi *et al.* (2008)	30	89.0	72.2–97.4	14.21
		Mgbakor *et al.* (2011)	24	29.2	12.6–51.1	14.07
		Oluwafemi and Oluwole (2012)	100	2.0	0.2–7.0	14.64
		Olaoye and Onilude (2009)	92	13.0	6.9–21.7	14.62
		Ugochukwu *et al.* (2015)	20	40.0	19.1–63.9	13.94
		Ugochukwu *et al.* (2015)	20	40.0	19.1–63.9	13.94
		Pooled prevalence (random effects)	364	40.0	12.4–71.7	100.00
		Test for heterogeneity				
		*Q*	225.7357	*I*^2^ (inconsistency)	97.34%	
		DF	6	95% CI for *I*^2^	96.05–98.21	
		Significance level	*p* < 0.0001			
*Salmonella* spp.	Borehole/well water	Akinyemi *et al.* (2011)	37	10.8	3.0–25.4	13.90
		Akinyemi *et al.* (2011)	37	13.5	4.5–28.8	13.90
		Akinyemi *et al.* (2011)	37	24.3	11.8–41.2	13.90
		Akinyemi *et al.* (2011)	37	21.6	9.8–38.2	13.90
		Akinyemi *et al.* (2011)	60	35.0	23.1–48.4	16.62
		Akinyemi *et al.* (2011)	37	8.1	1.7–21.9	13.90
		Akinyemi *et al.* (2011)	37	21.6	9.8–38.2	13.90
		Pooled prevalence (random effects)	282	19.8	13.1–27.4	100.00
		Test for heterogeneity				
		*Q*	14.4215	*I*^2^ (inconsistency)	58.40%	
		DF	6	95% CI for *I*^2^	3.93–81.98	
		Significance level	*p* = 0.0253			
*Staphylococcus* spp.	Raw/fresh milk	Dayok *et al.* (2019)	17	11.8	1.5–36.4	15.38
		Adesina *et al.* (2011)	15	26.0	7.4–54.4	14.08
		Olufemi *et al.* (2018)	64	14.1	6.6–25.0	33.12
		Aliyu *et al.* (2020)	14	7.1	0.2–33.9	13.40
		Aliyu *et al.* (2020)	34	2.9	0.07–15.3	24.02
		Pooled prevalence (random effects)	144	**12.3**	**6.3**–**20.0**	100.00
		Test for heterogeneity				
		*Q*	6.0808	*I*^2^ (inconsistency)	34.22%	
		DF	4	95% CI for *I*^2^	0.0–75.20	
		Significance level	*p* = 0.1932			
	*Nono/nunu*	Okonkwo (2011)	200	28.0	21.9–34.8	14.90
		Dafur *et al.* (2018)	300	16.3	12.3–21.0	14.97
		Fowoyo and Ogunbanwo (2016)	54	88.9	77.4–95.8	14.31
		Usman and Mustapha (2016)	140	2.9	0.8–7.1	14.80
		Usman and Mustapha (2016)	140	13.6	8.4–20.4	14.80
		Yabaya *et al.* (2012)	10	100.0	69.2–100.0	11.77
		Aliyu *et al.* (2020)	66	6.1	1.7–14.8	14.45
		Pooled prevalence (random effects)	910	32.6	14.7–53.8	100.00
		Test for heterogeneity				
		*Q*	235.8627	*I*^2^ (inconsistency)	97.46%	
		DF	6	95% CI for *I*^2^	96.24–98.28	
		Significance level	*p* < 0.0001			
*Escherichia* spp.	*Nono/nunu*	Okonkwo (2011)	200	43.0	36.0–50.2	7.96
		Enabulele and Nwankiti (2016)	200	26.5	20.5–33.2	7.96
		Enabulele and Nwankiti (2016)	200	24.0	18.3–30.5	7.96
		Enabulele and Nwankiti (2016)	200	40.5	33.6–47.7	7.96
		Enabulele and Nwankiti (2016)	200	43.0	36.0–50.2	7.96
		Enabulele *et al.* (2015)	200	26.5	20.5–33.2	7.96
		Enabulele *et al.* (2015)	200	24.0	18.3–30.5	7.96
		Enabulele *et al.* (2015)	200	40.5	33.6–47.7	7.96
		Enabulele *et al.* (2015)	200	43.0	36.0–50.2	7.96
		Dafur *et al.* (2018)	300	43.0	37.3–48.8	8.06
		Enem *et al.* (2015)	127	1.6	0.2–5.6	7.79
		Yabaya *et al.* (2012)	10	90.0	55.5–99.7	4.81
		Yakubu *et al.* (2018)	100	2.0	0.2–7.0	7.67
		Pooled prevalence (random effects)	2337	30.7	21.9–40.2	100.00
		Test for heterogeneity				
		*Q*	281.8939	*I*^2^ (inconsistency)	95.74%	
		DF	12	95% CI for *I*^2^	94.10–96.93	
		Significance level	*p* < 0.0001			

CI, confidence interval; DF, degrees of freedom.

## Discussion

This review identified beverage types consumed in Nigeria ranging from traditional, nonindustrial, to processed alcoholic and nonalcoholic drinks. However, the five most studied beverages were sachet water, borehole/well water, cereal-based beverages, raw/fresh milk, and *nono/nunu*. Sachet and borehole water are important sources of potable drinking water in Nigeria. The affordability and availability of sachet water make it the primary source of potable water for most of the Nigerian populace (Izah *et al.*, 2016). A survey conducted by Dada and Awotunde (2017) noted a consumer preference for home-prepared beverages as opposed to carbonated drinks. They reported a preference for fruit juice and *kunun-zaki* (cereal-based beverage). *Nono* constitutes part of the crucial staple food for the nomadic Fulani, of Northern Nigeria, and its consumption, together with that of raw milk and milk by-products, is popular among the rural and urban population, a preference that is partly attributed to a belief that it is more nutritious than pasteurized milk products (Egwaikhide *et al.*, 2014).

The spatial assessment revealed an unequal distribution of studies on beverage-associated hazards in Nigeria. This difference may suggest different research priorities by research institutions within these states. It could also imply inadequate resource allocation, to support research, by the states. This is likely the case in neighboring countries given the report by the Global Food Safety Partnership, which highlights the fact that investments that support food safety work are lacking in sub-Saharan Africa, especially in the dominant informal markets (Global Food Safety Partnership, 2019). The paucity of information on the hazards in beverages from Adamawa, Yobe, Jigawa, Katsina, Kwara, Kogi, Kebbi, and Zamfara states may be indicative of underreporting, or reduced research prioritization in the areas. Notable is that each state in Nigeria has at least two universities (Mogaji, 2019). With the growing interest in food safety, regionally and globally, it is expected that more research will be undertaken to generate evidence on current gaps and effects of interventions.

It was found that much of the literature identified, which was otherwise eligible for review, was deemed of poor quality (130/256) and therefore excluded from this literature review, indicating a potential capacity gap in research design and scientific writing. Limitations in resources, infrastructure, and training have been highlighted as barriers to the generation of high-quality scientific publications in Nigeria and several other African countries (Ajao and Ugwu, 2011; Kumwenda *et al.*, 2017).

This review identified studies on a wide range of biological hazards with records on bacterial hazards constituting the bulk (64%) with genera *Escherichia*, *Staphylococcus*, *Salmonella*, and *Klebsiella* being the most common bacterial pathogens studied. This agrees with a review in selected African countries where *Escherichia coli*, *Salmonella* spp., and *Staphylococcus aureus* were the most frequently studied pathogens in ready-to-eat foods and beverages in the selected studies (Paudyal *et al.*, 2017). These pathogens have been widely associated with foodborne outbreaks and sporadic cases of foodborne toxicity in humans (Ivbade *et al.*, 2014). Characterization of *E. coli* into pathotypes was done in 17/78 records, all reporting enterohemorrhagic *E. coli* in milk and milk products. These pathogens are important causes of diarrhea especially in children younger than 5 years (Havelaar *et al.*, 2015). The pooled prevalence estimate of *Staphylococcus* spp. in raw/fresh milk derived from this study may be useful in complementing other baseline data in milk-safety interventions in Nigeria.

This review revealed a paucity of studies incriminating *Campylobacter* spp., *Vibrio* spp., and *Shigella* spp. as contaminants in beverages yet they are major contributors to foodborne disease burden and have been ranked among the top 10 priority hazards in Africa (Havelaar *et al.*, 2015). *Vibrio cholerae*, the causal agent of cholera, is a significant cause of mortality associated with the consumption of contaminated water. Together with *Shigella* spp. and *Campylobacter* spp., they are common contaminants of water sources in developing countries, stemming from pollution by animal and human feces (Cabral, 2010; Thomas *et al.*, 2020).

Fungi and their metabolites were also reported in beverages. Genera *Aspergillus*, *Penicillium*, and *Rhizopus* were the commonest reported in this review. *Aspergillus* spp. was found to be the most abundant fungus based on pooled prevalence estimates, and high contamination rates were detected in traditional nonalcoholic beverages. Aspergillosis results in symptoms ranging from allergic reactions, saprophytic lung disease, and otomycosis to a systemic disease characterized by immune system failure (Barnes and Marr, 2006). Aspergillosis poses a serious diagnostic and management challenge, hence a threat to the public (Garbino, 2004; Barnes and Marr, 2006). *Rhizopus* spp. and *Penicillium* spp. are important opportunistic pathogens. Their presence additionally introduces the risk of food intoxication and spoilage. Fungal metabolites such as zearalenone, fumonisin, and acetyl-deoxynivalenol were studied and reported. Mycotoxins produce acute to chronic long-term health effects, varying from enteric disease to induction of cancers and immune deficiency (Onuorah and Odibo, 2018). Aflatoxins whose burden was found to be highest in the AFR D subregion (Havelaar *et al.*, 2015) are a particular concern to children who are weaned primarily on cereal (AFB1) and milk-based products (AFM1).

The relatively small number of studies on mycotoxins may present a research gap, considering the vulnerability of the production process of traditional alcoholic beverages to contamination by toxigenic fungi (Olaniyi and Akinyele, 2020). According to Adeloye *et al.* (2019), Nigeria has a high prevalence of alcohol abuse and high consumption of cereal-based alcoholic beverages. This is therefore an area of public health concern. Similarly, the occurrence of parasitic contamination of beverages was scantily documented as a meager 13 records were identified, with all reporting positive findings, despite utilizing relatively insensitive detection techniques (microscopy) (Stensvold and Nielsen, 2012). *Ascaris* spp., *Giardia* spp., and *Entamoeba* spp. were the most common parasites reported from sachet water. Sachet water is a crucial source of drinking water, consumed by over 120 million Nigerians (Izah *et al.*, 2016); this population is potentially at risk of enteric disease caused by parasites, and therefore, quality issues are of significant concern. These parasites have been estimated to account for the ill-health of 68 million people and 765,000 foodborne DALYs in a year (Havelaar *et al.*, 2015). Notably, of the biological hazards identified, no viral pathogens have been reported. This is despite a significant burden from viral hazards, including norovirus (2.5 million DALYs and 35,000 deaths per year, globally), which can be transmitted through contaminated water (Havelaar *et al.*, 2015).

Chemical contaminants, particularly heavy metals, were reported in beverages at levels above the recommended level by the Nigerian regulatory authorities and the WHO. The reported occurrences of heavy metal contamination suggest a growing concern about the potability of beverages consumed in Nigeria. Heavy metals are toxic to animals, humans, and the environment. Due to their toxicity and potential bioaccumulation, serious and mandatory monitoring especially in food should be implemented (Morais *et al.*, 2012). Water is a fundamental element in food processing and a key constituent in the production and preparation process of beverages, including cleaning and sterilization of equipment, and quality issues in water are therefore likely to be transferred to other food. Mogborukor (2012) attributes the decline in potability in Nigeria to environmental deterioration brought about by the increase in population and urbanization. The chemical hazards outlined here have significant health effects, including brain damage, liver damage, kidney damage, enteric disease, damage to the reproductive system, anemia, cancer, osteopathy, oxidative stress, and oxidation of biological molecules among others (Izah *et al.*, 2016; Engwa *et al.*, 2019).

Food hygiene and safety management systems (FHSM) in many developing African economies are still in their infancy. Its progression has been greatly impeded by the absence or/and underutilization of economic data on the impact of foodborne diseases (Akhtar *et al.*, 2014). Subsequently, there is low prioritization of food safety concerns evidenced by scanty food safety policies, weak supportive legislative environment, and underreporting of foodborne disease outbreaks (Akhtar *et al.*, 2014). Among the components of FHSM is a dynamic and robust risk analysis, which entails hazard identification, characterization, determination of the level of occurrence in food, and exposure assessment among others (Iro *et al.*, 2020). This review outlines deficiencies in quality research outputs at this level. The gaps identified are in alignment with the report by Iro *et al.* (2020) on food safety and management in Nigeria. Iro *et al.* (2020) suggest an integrated approach to Nigeria's food safety concerns, involving the adoption of a Hazard Analysis Critical Control Points (HACCP) system.

The limited capacity of food control laboratories in Africa significantly weakens the surveillance infrastructure both at the national and subnational levels (Akhtar *et al.*, 2014). The main causes include inadequate funding, lack of equipment and personnel, lack of recurrent expenditure to facilitate maintenance of equipment and replenish disposables, and inadequate quality assurance procedures (FAO/WHO, 2005). Developing economies are urged to allocate resources for establishing effective food safety management systems. Accurate, timely, and pertinent information is paramount to compelling policymakers to prioritize investments in food safety systems. Integrated efforts led by research and academic institutes, and encompassing other stakeholders in food safety in a “One Health” framework, should ensure that policymakers receive reliable information on the economic and health implications of food safety management systems and on the measures required to attain quality data (FAO/WHO, 2005; FAO, 2011).

## Conclusion

This review has highlighted the presence of several hazards of high importance to public health in commonly consumed beverages in Nigeria and an apparently low investment in the investigation of other significant foodborne hazards known to occur in the African region. The data presented here provide an entry point for future quantitative risk assessments both to determine the level of exposure of the community to these and other hazards, and also for the identification of the most effective mitigation strategies to reduce these risks and improve health outcomes in Nigeria. In addition, the disparity in both the geographic scope and the low consideration of other important hazards may be a useful input in the national foodborne surveillance planning.

## Supplementary Material

Supplemental data

## Data Availability

The data are not publicly available but can be provided on request.
