# Supplementary material for: Assessment of Foodborne Disease Hazards in Beverages Consumed in Nigeria: A Systematic Literature Review
Source: Foodborne Pathog Dis. 2022 Jan 11;19(1):1–18. doi: 10.1089/fpd.2021.0043 (PMC8785768; doi:10.1089/fpd.2021.0043)
Supplement: Supplemental data [file Supp_DataS1.docx]

**SUPPLEMENTARY MATERIALS**

**Supplementary 1**

Search syntaxes for ScienceDirect, Google Scholar and PubMed

1. SCIENCE DIRECT SEARCH OUTPUTS (8^th^ June 2020)

|  | **ScienceDirect Syntaxes** | **Search output** |
| --- | --- | --- |
| 1 | Beverage AND (safety OR disease OR pathogen OR poison OR microbe OR Toxin OR chemical) AND Nigeria | 2072 |
| 2 | Milk AND (safety OR disease OR pathogen OR poison OR microbe OR Toxin OR chemical) AND Nigeria | 4252 |
| 3 | “Drinking water” AND (safety OR disease OR pathogen OR poison OR microbe OR Toxin OR chemical) AND Nigeria | 2806 |
| 4 | Drink AND (safety OR disease OR pathogen OR poison OR microbe OR Toxin OR chemical) AND Nigeria | 5436 |
| 5 | “Potable water”AND (safety OR disease OR pathogen OR poison OR microbe OR Toxin OR chemical) AND Nigeria | 638 |
| 6 | Juice AND (safety OR disease OR pathogen OR poison OR microbe OR Toxin OR chemical) AND Nigeria | 1883 |
| 7 | Alcohol AND (safety OR disease OR pathogen OR poison OR microbe OR Toxin OR chemical) AND Nigeria | 6155 |
| 8 | Nonalcoholic AND (safety OR disease OR pathogen OR poison OR microbe OR Toxin OR chemical) AND Nigeria | 179 |
| 9 | Soymilk AND (safety OR disease OR pathogen OR poison OR microbe OR Toxin OR chemical) AND Nigeria | 74 |
| 10 | Kunun-zaki AND (safety OR disease OR pathogen OR poison OR microbe OR Toxin OR chemical) AND Nigeria | 30 |
| 11 | Kunu AND (safety OR disease OR pathogen OR poison OR microbe OR Toxin OR chemical) AND Nigeria | 38 |
| 12 | Zobo AND (safety OR disease OR pathogen OR poison OR microbe OR Toxin OR chemical) AND Nigeria | 18 |
| 13 | kunlun AND (safety OR disease OR pathogen OR poison OR microbe OR Toxin OR chemical) AND Nigeria | 16 |
| 14 | Nono AND (safety OR disease OR pathogen OR poison OR microbe OR Toxin OR chemical) AND Nigeria | 70 |
| 15 | Ogwo AND (safety OR disease OR pathogen OR poison OR microbe OR Toxin OR chemical) AND Nigeria | 4 |
| 16 | Soborodo AND (safety OR disease OR pathogen OR poison OR microbe OR Toxin OR chemical) AND Nigeria | 3 |
| 17 | Kurumaya AND (safety OR disease OR pathogen OR poison OR microbe OR Toxin OR chemical) AND Nigeria | 1 |
| 18 | Isaya AND (safety OR disease OR pathogen OR poison OR microbe OR Toxin OR chemical) AND Nigeria | 3 |
| 19 | Kindirmo AND (safety OR disease OR pathogen OR poison OR microbe OR Toxin OR chemical) AND Nigeria | 3 |
| 20 | Soyamilk AND (safety OR disease OR pathogen OR poison OR microbe OR Toxin OR chemical) AND Nigeria | 1 |

| 21 | Beverage AND (borne OR associated OR illness OR pathogen OR virus OR parasite) AND Nigeria | 1957 |
| --- | --- | --- |
| 22 | Milk AND (borne OR associated OR illness OR pathogen OR virus OR parasite) AND Nigeria | 4,078 |
| 23 | Drink AND (borne OR associated OR illness OR pathogen OR virus OR parasite) AND Nigeria | 5389 |
| 24 | “Drinking water” AND (borne OR associated OR illness OR pathogen OR virus OR parasite) AND Nigeria | 2706 |
| 25 | “Potable water” AND (borne OR associated OR illness OR pathogen OR virus OR parasite) AND Nigeria | 600 |
| 26 | Juice AND (borne OR associated OR illness OR pathogen OR virus OR parasite) AND Nigeria | 1663 |
| 27 | Kunlun AND (borne OR associated OR illness OR pathogen OR virus OR parasite) AND Nigeria | 16 |
| 29 | Alcohol AND (borne OR associated OR illness OR pathogen OR virus OR parasite) AND Nigeria | 5910 |
| 29 | Nonalcoholic AND (borne OR associated OR illness OR pathogen OR virus OR parasite) AND Nigeria | 169 |
| 30 | Soymilk AND (borne OR associated OR illness OR pathogen OR virus OR parasite) AND Nigeria | 59 |
| 31 | Kunun-zaki AND (borne OR associated OR illness OR pathogen OR virus OR parasite) AND Nigeria | 26 |
| 32 | Kunu AND (borne OR associated OR illness OR pathogen OR virus OR parasite) AND Nigeria | 32 |
| 33 | Nono AND (borne OR associated OR illness OR pathogen OR virus OR parasite) AND Nigeria | 65 |
| 34 | Zobo AND (borne OR associated OR illness OR pathogen OR virus OR parasite) AND Nigeria | 16 |
| 35 | Kurumaya AND (borne OR associated OR illness OR pathogen OR virus OR parasite) AND Nigeria | 1 |
| 36 | Isaya AND (borne OR associated OR illness OR pathogen OR virus OR parasite) AND Nigeria | 4 |
| 37 | Soborodo AND (borne OR associated OR illness OR pathogen OR virus OR parasite) AND Nigeria | 3 |
| 38 | Ogwo AND (borne OR associated OR illness OR pathogen OR virus OR parasite) AND Nigeria | 5 |
| 39 | Soyamilk AND (borne OR associated OR illness OR pathogen OR virus OR parasite) AND Nigeria | 1 |
| 40 | Kindirmo AND (borne OR associated OR illness OR pathogen OR virus OR parasite) AND Nigeria | 3 |

1. GOOGLE SCHOLAR SEARCH OUTPUTS (5^th^ June 2020)

|  | **Google Scholar Syntax** | **Hits** | **Records Extracted** |
| --- | --- | --- | --- |
| 1 | Nigeria AND milk safety\|borne\|related\|associated\|illness\|disease\|pathogen\|poison\|microbe\|virus\|parasite\|toxin\|toxicant\|metabolite\|chemical\|intoxication –"breast milk" –"human milk" | 56200 | 300 |
| 2 | Nigeria AND drinking water safety\|borne\|related\|associated\|illness\|disease\|pathogen\|poison\|microbe\|virus\|parasite\|toxin \|toxicant\|metabolite\|chemical\|intoxication –"breast milk" –"human milk" | 65,600 | 300 |
| 3 | Nigeria AND potable water safety\|borne\|related\|associated\|illness\|disease\|pathogen\|poison\|microbe\|virus\|parasite\|toxin \|toxicant\|metabolite\|chemical\|intoxication –"breast milk" –"human milk" | 19,900 | 300 |
| 4 | Nigeria AND drink safety\|borne\|related\|associated\|illness\|disease\|pathogen\|poison\|microbe\|virus\|parasite\|toxin \|toxicant\|metabolite\|chemical\|intoxication –"breast milk" –"human milk" | 28700 | 300 |
| 5 | Nigeria AND beverage safety\|borne\|related\|associated\|illness\|disease\|pathogen\|poison\|microbe\|virus\|parasite\|toxin \|toxicant\|metabolite\|chemical\|intoxication –"breast milk" –"human milk" | 18600 | 300 |
| 5 | Nigeria AND juice safety\|borne\|related\|associated\|illness\|disease\|pathogen\|poison\|microbe\|virus\|parasite\|toxin \|toxicant\|metabolite\|chemical\|intoxication –"breast milk" –"human milk" | 19400 | 300 |
| 7 | Nigeria AND soy milk safety\|borne\|related\|associated\|illness\|disease\|pathogen\|poison\|microbe\|virus\|parasite\|toxin \|toxicant\|metabolite\|chemical\|intoxication –"breast milk" –"human milk" | 8770 | 300 |
| 8 | Nigeria AND soya milk safety\|borne\|related\|associated\|illness\|disease\|pathogen\|poison\|microbe\|virus\|parasite\|toxin \|toxicant\|metabolite\|chemical\|intoxication –"breast milk" –"human milk" | 18500 | 300 |
| 9 | Nigeria AND "kunun-zaki"safety\|borne\|related\|associated\|illness\|disease\|pathogen\|poison\|microbe\|virus\|parasite\|toxin \|toxicant\|metabolite\|chemical\|intoxication –"breast milk" –"human milk" | 566 | 300 |
| 10 | Nigeria AND kindirmo safety\|borne\|related\|associated\|illness\|disease\|pathogen\|poison\|microbe\|virus\|parasite\|toxin \|toxicant\|metabolite\|chemical\|intoxication –"breast milk" –"human milk" | 65 | 93 |
| 11 | Nigeria AND kunlun safety\|borne\|related\|associated\|illness\|disease\|pathogen\|poison\|microbe\|virus\|parasite\|toxin \|toxicant\|metabolite\|chemical\|intoxication –"breast milk" –"human milk" | 352 | 300 |
| 12 | Nigeria AND kunu safety\|borne\|related\|associated\|illness\|disease\|pathogen\|poison\|microbe\|virus\|parasite\|toxin \|toxicant\|metabolite\|chemical\|intoxication –"breast milk" –"human milk" | 1030 | 300 |
| 13 | Nigeria AND soborodo safety\|borne\|related\|associated\|illness\|disease\|pathogen\|poison\|microbe\|virus\|parasite\|toxin \|toxicant\|metabolite\|chemical\|intoxication –"breast milk" –"human milk" | 220 | 232 |
| 14 | Nigeria AND nono safety\|borne\|related\|associated\|illness\|disease\|pathogen\|poison\|microbe\|virus\|parasite\|toxin \|toxicant\|metabolite\|chemical\|intoxication –"breast milk" –"human milk" | 18000 | 300 |
| 15 | Nigeria AND zobo safety\|borne\|related\|associated\|illness\|disease\|pathogen\|poison\|microbe\|virus\|parasite\|toxin \|toxicant\|metabolite\|chemical\|intoxication –"breast milk" –"human milk" | 850 | 300 |
| 16 | Nigeria AND kurumaya safety\|borne\|related\|associated\|illness\|disease\|pathogen\|poison\|microbe\|virus\|parasite\|toxin \|toxicant\|metabolite\|chemical\|intoxication –"breast milk" –"human milk" | 7 | 7 |
| 17 | Nigeria AND ogwo safety\|borne\|related\|associated\|illness\|disease\|pathogen\|poison\|microbe\|virus\|parasite\|toxin \|toxicant\|metabolite\|chemical\|intoxication –"breast milk" –"human milk" | 1250 | 300 |
| 18 | Nigeria AND isaya safety\|borne\|related\|associated\|illness\|disease\|pathogen\|poison\|microbe\|virus\|parasite\|toxin \|toxicant\|metabolite\|chemical\|intoxication –"breast milk" –"human milk" | 258 | 299 |
| 19 | Nigeria AND non-alcoholic safety\|borne\|related\|associated\|illness\|disease\|pathogen\|poison\|microbe\|virus\|parasite\|toxin \|toxicant\|metabolite\|chemical\|intoxication –"breast milk" –"human milk" | 6980 | 300 |
| 20 | Nigeria AND alcohol safety\|borne\|related\|associated\|illness\|disease\|pathogen\|poison\|microbe\|virus\|parasite\|toxin \|toxicant\|metabolite\|chemical\|intoxication –"breast milk" –"human milk" | 103,000 | 300 |
| **PubMed Syntax (13^th^ May 2020)** | | | Search output |
| milk or “drinking water” or “potable water” or drink* or beverage* or juice* or soymilk or soyamilk or "kunun-zaki" or kindirmo or kunlun or kunu or soborodo or nono or zobo or kurumaya or ogwo or isaya or kunu or "non-alcoholic" or "nonalcoholic” or alcohol*) AND (safety OR borne OR related OR associated OR illness OR disease OR pathogen OR poison* OR microb* OR virus* OR parasit* OR Toxin OR toxicant OR metabolite OR chemical OR intoxica*) AND Nigeria* NOT “breast milk” NOT "breast-milk" NOT "breastmilk" | | | 1315 |
